# Whole-genome genotyping and resequencing reveal the association of a deletion in the complex interferon alpha gene cluster with hypothyroidism in dogs

**DOI:** 10.1186/s12864-020-6700-3

**Published:** 2020-04-16

**Authors:** Matteo Bianchi, Nima Rafati, Åsa Karlsson, Eva Murén, Carl-Johan Rubin, Katarina Sundberg, Göran Andersson, Olle Kämpe, Åke Hedhammar, Kerstin Lindblad-Toh, Gerli Rosengren Pielberg

**Affiliations:** 1 Science for Life Laboratory, Department of Medical Biochemistry and Microbiology, Uppsala University, Uppsala, Sweden; 2 Department of Animal Breeding and Genetics, Swedish University of Agricultural Sciences, Uppsala, Sweden; 3 Department of Medicine (Solna), Karolinska Institutet, Stockholm, Sweden; 4 Department of Clinical Sciences, Swedish University of Agricultural Sciences, Uppsala, Sweden; 5 Broad Institute of MIT and Harvard, Cambridge, MA USA

**Keywords:** Dog, Hypothyroidism, Genome-wide association study, Fine-mapping, Whole-genome sequencing, Long-read sequencing, Type I interferon genes

## Abstract

**Background:**

Hypothyroidism is a common complex endocrinopathy that typically has an autoimmune etiology, and it affects both humans and dogs. Genetic and environmental factors are both known to play important roles in the disease development. In this study, we sought to identify the genetic risk factors potentially involved in the susceptibility to the disease in the high-risk Giant Schnauzer dog breed.

**Results:**

By employing genome-wide association followed by fine-mapping (top variant *p*-value = 5.7 × 10^− 6^), integrated with whole-genome resequencing and copy number variation analysis, we detected a ~ 8.9 kbp deletion strongly associated (*p*-value = 0.0001) with protection against development of hypothyroidism. The deletion is located between two predicted Interferon alpha (*IFNA*) genes and it may eliminate functional elements potentially involved in the transcriptional regulation of these genes. Remarkably, type I IFNs have been extensively associated to human autoimmune hypothyroidism and general autoimmunity. Nonetheless, the extreme genomic complexity of the associated region on CFA11 warrants further long-read sequencing and annotation efforts in order to ascribe functions to the identified deletion and to characterize the canine *IFNA* gene cluster in more detail.

**Conclusions:**

Our results expand the current knowledge on genetic determinants of canine hypothyroidism by revealing a significant link with the human counterpart disease, potentially translating into better diagnostic tools across species, and may contribute to improved canine breeding strategies.

## Background

The domestic dog has proven to be an effective animal model to identify genetic risk factors underlying phenotypic traits and diseases shared with humans, as demonstrated by several successful studies in recent years [1–4]. Dogs spontaneously develop a wide range of immune-mediated, endocrine and cardiovascular disorders, as well as cancers and nervous system diseases [5, 6]. One of the most common endocrinopathies affecting both dogs and humans is hypothyroidism [7, 8]. In both species, symptoms are typically non-specific and include weight gain, tiredness, alopecia and impaired hair quality, as well as intolerance to cold. This demonstrates that thyroid hormones are master regulators of metabolism, highlighting their importance in steering vital body functions [9, 10].

Excluding rare congenital hypothyroidism and other sporadic thyroid-related disorders, autoimmune hypothyroidism accounts for most cases in which this gland fails to produce sufficient amount of its specific hormones, i.e. thyroxine (T4) and triiodothyronine (T3) [11, 12]. In the western countries, where the daily intake of iodine is sufficient, autoimmune Hashimoto’s thyroiditis (HT) represents the major cause of human hypothyroidism [13]. The canine equivalent of HT is called canine lymphocytic thyroiditis (CLT) and it is characterized by a progressive degeneration of the thyroid gland and its function, with presence of circulating autoantibodies against thyroglobulin (TgAA) and infiltration of B and T lymphocytes into the thyroid [14–17].

The Beagle, Boxer, Dobermann Pinscher, English Setter, Gordon Setter, Giant Schnauzer, Hovawart, Old English Sheepdog and the Rhodesian Ridgeback are among the dog breeds showing increased risk of developing hypothyroidism [14, 18–22]. Moreover, the disease shows clear clustering within pedigrees in these breeds [23]. Overall, this clearly suggests the presence of heritable genetic components increasing the risk of developing the disease. According to a Swedish epidemiological survey regarding hypothyroidism susceptibility in different dog breeds, Giant Schnauzer appeared as a high-risk breed, with a six-fold increased risk compared to the general dog population [19]. This was confirmed by another study that estimated the prevalence of hypothyroidism in the Swedish population of Giant Schnauzer dogs to as high as ~ 16% [24].

Determining the genetic etiology of hypothyroidism is of major interest due to the high prevalence and the impact of the disease in both humans and dogs. In previous genetic studies aimed at mapping this disease in high-risk dog breeds, Kennedy and colleagues [25], as well as Wilbe and colleagues [26], employed a candidate gene approach and found associations with dog leukocyte antigen (DLA) class II alleles. More recently, by using an integrated three high-risk breed genome-wide association and meta-analysis approach our group detected a risk locus, shared by multiple breeds, in a region of CFA12 not harboring the DLA [1]. However, neither the entire underlying risk in specific breeds nor the genetic susceptibility shared among dog breeds can be fully explained by the hitherto identified disease-associated alleles. This suggests the existence of additional genetic risk factors, thus confirming the proposed complex etiology of canine hypothyroidism.

Here we sought to identify additional genetic loci potentially involved in disease susceptibility in dogs. To tackle this challenge, we employed genome-wide association (GWA) analysis followed by a fine-mapping approach using breed-specific variants detected by whole-genome resequencing, as well as copy number variation (CNV) analysis in a high-risk Giant Schnauzer breed. In this study we expand the current knowledge about canine hypothyroidism and its genetic determinants by describing a novel locus associated with the development of this disease. The identification of this locus implicates a noteworthy link with the human counterpart of the disease, thus confirming the validity of employing the domestic dog as a disease animal model.

## Results

### GWA analysis identifies a 8.9 Mbp protective haplotype on CFA11

We genotyped 115 Giant Schnauzer dogs (*n*_cases_ = 73, *n*_controls_ = 42) using ~ 170,000 markers (Illumina 170 K CanineHD BeadChip), and subsequently performed a GWA analysis of hypothyroidism using the markers and individuals passing through the data quality control (QC) and filtering steps (*n*_case__s_ = 71, *n*_controls_ = 36). The multidimensional scaling (MDS) plot generated in the individual-based QC step highlighted the presence of six outlier individuals, which were subsequently discarded (Fig. S1a). A thorough examination of the phenotypic data revealed that five of the outlying samples had a different coat colour compared with the rest of the dogs, resulting in a separate cluster. Moreover, two additional samples showed sex discrepancies between phenotypic and genetic data. Out of the ~ 170,000 single nucleotide polymorphisms (SNPs) genotyped, 112,683 passed the marker-based QC. Furthermore, our study cohort did not show any sex bias between the two phenotypic groups (*p*-value = 0.2, phi coefficient = 0.1).

Case and control dogs did not form separate clusters on the MDS plot generated using the pruned dataset, thus suggesting absence of population stratification in our cohort (Fig. S1b). The association analysis performed using a mixed model, correcting for population structure and cryptic relatedness, consistently showed no inflation (λ = 0.93), as displayed in the quantile-quantile (QQ) plot (Fig. S2a). However, the genomic inflation factor λ showed some degrees of deflation. The QQ plot also depicts the statistical significance levels (see Methods, section “Genome-wide association analysis”).

**Fig. 1:**
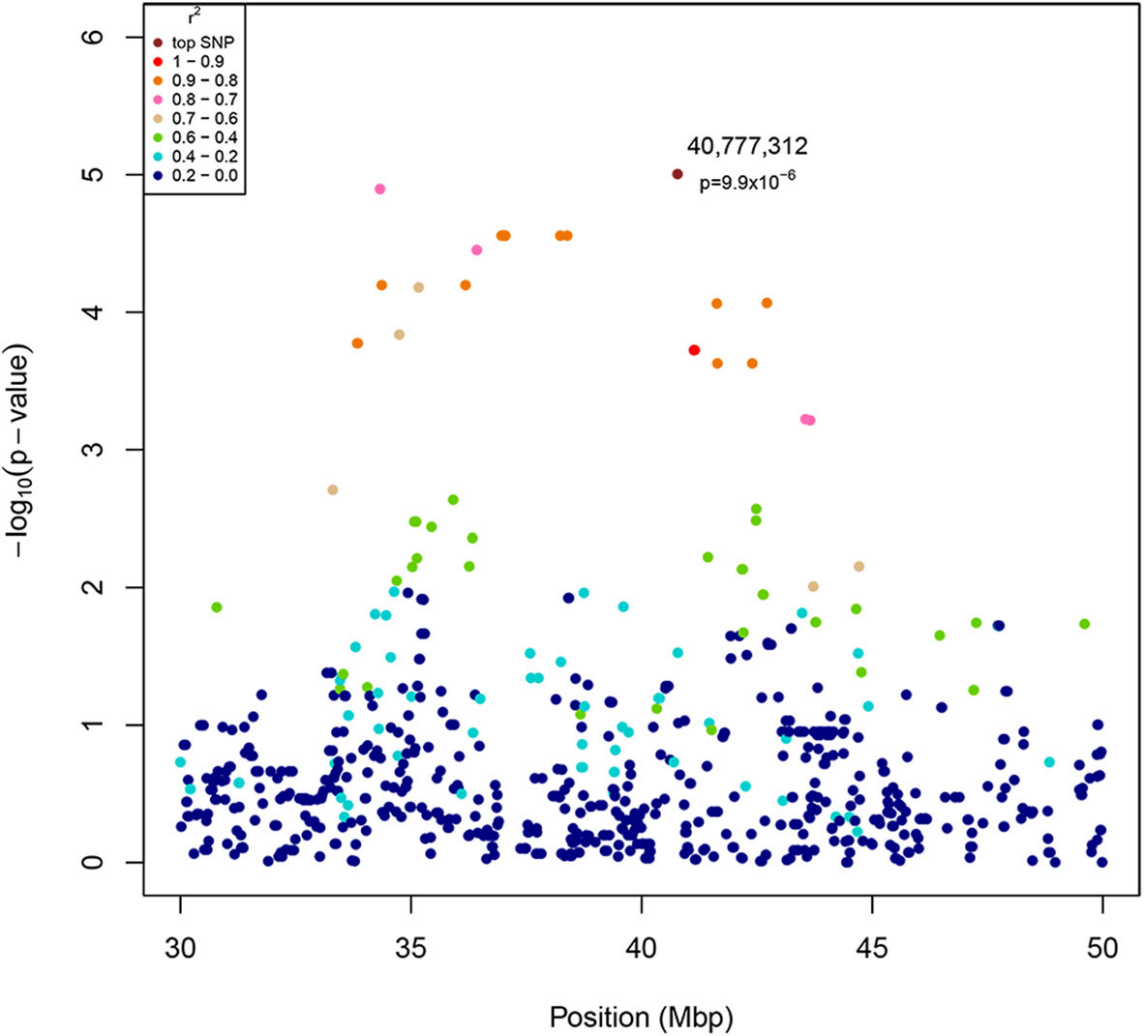
LD Manhattan plot showing a zoom to the candidate region on CFA11. The plot indicates r^2^ values of each SNP in respect to the GWA analysis top SNP

Based on the 95% empirical SNP distribution confidence intervals (CI_95_), we found a suggestive genetic association to a region on CFA11. The top SNP was located at CanFam3.1 genomic coordinate CFA11: 40,777,312 (*p*-value_raw_ = 9.9 × 10^− 6^; odds ratio (OR) = 0.15, CI_OR_ = 0.05–0.39) (Fig. S2b). The minor allele frequency (MAF) of the top SNP was 0.12 across all samples, whereas 0.05 in cases and 0.26 in controls. The odds ratio (OR) of the top SNP suggests that the associated locus is protective in this breed. The candidate locus was defined as spanning 8.9 Mbp (33,834,431 – 42,717,190 bp) (Fig. 1), based on pairwise linkage disequilibrium (LD) estimates (r^2^ ≥ 0.8) of the top SNP to the rest of the SNPs on CFA11. Conditional analysis confirmed the independence of the association signal (Fig. S3). The associated region includes more than 30 genes according to the improved canine genome annotation [27] and the canine RefSeq annotation [28]. Among these genes there are many obvious candidates with potential roles in immune response and immune system regulation.

### Fine-mapping narrows down the candidate region to 4.18 Mbp

We selected two case and one control samples for high coverage (HC) Illumina short-read whole-genome resequencing (WGS). For these samples we generated an average of 46X genome coverage (SD = 2.9). Moreover, the genomes of 10 case and 10 control samples were sequenced at low coverage (LC) with the same technology, generating an average of 6.8X genome coverage per individual (SD = 1.0). More than 92% (SD = 0.4) of the reads aligned to the dog reference genome in both groups of resequenced samples (Table S1).

In the HC samples we identified 18,470 SNPs in the extended associated interval (33,000,000 - 43,000,000 bp) on CFA11. In the first pruning step we removed 5264 variants with identical genotypes between the two HC cases and the HC control. The remaining 13,206 SNPs were screened in order to identify a subset of SNPs with functional potential covering the whole region of association. The selection of SNPs with functional potential (*n* = 740) was genotyped using Sequenom MassARRAY in 96 dogs out of the initial GWA analysis cohort of 107 individuals, leaving out 11 individuals due to poor quality or lack of DNA specimen. Genotyping success rate was 95.5% (33 out of 740 SNPs failed due to technical reasons), leaving 707 polymorphisms for further analyses (Table S2).

Among the 707 successfully regenotyped SNPs, we first discarded 69 monomorphic variants (MAF < 0.001) that likely represented false variant calls in the HC individuals. The remaining 638 SNPs were subsequently combined with the SNPchip variants covering the extended region of association, while discarding one of the duplicated control SNPs (i.e. those included in both the Ilumina SNPChip and the Sequenom MassARRAY experiments) (Table S2) based on lower variant call rate. Thereby we generated a reference set composed of 1110 SNPs that was used for imputation in the 11 excluded samples. Out of the total 7293 imputed genotypes, 4876 were retained for further analyses (67%) after the application of the imputation likelihood-based filters.

**Fig. 2:**
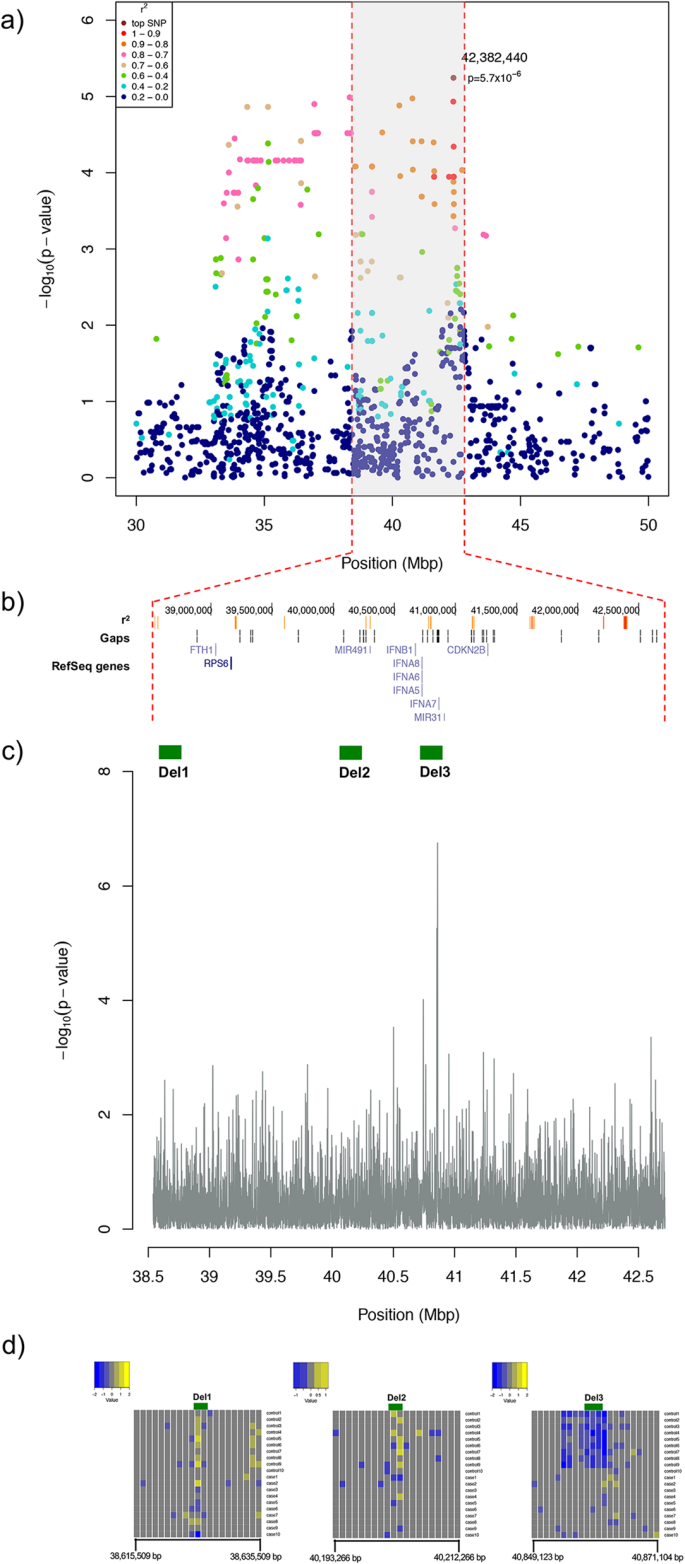
**a** LD Manhattan plot showing a zoom to the candidate region on CFA11. The plot indicates r^2^ values of each SNP in respect to the new GWA analysis top SNP. The fine-mapped region of association is highlighted with a grey shadow. **b** UCSC genome browser-based panel showing genomic location (bp), highly linked SNPs (r^2^ > 0.8) in the fine-mapped region colored according to their r^2^ (darker colors indicating higher LD) in respect to the fine-mapping top SNP, location of genomic gaps, and location of RefSeq protein coding genes. **c** Plot showing the –log10(*p*-value) of the difference in coverage between the LC case and control groups in the fine-mapped region. The green boxes represent the three CNVs (Del1, Del2 and Del3) predicted by CNVnator in the HC individuals. **d** Heatmaps showing fold coverage differences (M-value) between the LC case and control groups for the windows overlapping with Del1, Del2 and Del3, +/− 10 Kbs. The potential CNVs in the upstream region of Del3 were also predicted by CNVnator, but were discarded either because of overlap with a gap or repetitive sequences

The association test performed on the final and complete dataset, obtained by merging the filtered imputed data with the whole SNPChip and the regenotyping data, showed no inflation (λ = 0.97). Despite showing a slight degree of deflation, the inclusion of additional SNPs to the original genotypic data significantly improved the ratio between the observed and the theoretical SNPs *p*-value distribution (Fig. S4a). The statistical significance of the test largely exceeded the empirical CI_95_ levels, and was additionally suggestive towards the empirical genome-wide threshold calculated after 1000 permutations (*p*-value = 5.4 × 10^− 6^) (Fig. S4a). We could therefore confirm the detection of a statistically significant association to the same region of CFA11 with a new top SNP (fine-mapping top SNP) located at position 42,382,440 (*p*-value_raw_ = 5.7 × 10^− 6^; OR = 0.07, CI_OR_ = 0.01–0.28) (Fig. S4b). The MAF of the fine-mapping top SNP was 0.09 in the whole sample set, with 0.02 and 0.22 in cases and controls, respectively. This SNP lies in a conserved element with a SiPhy-omega LOD-score equal to 7.4, and a SiPhy-pi LOD-score equal to 7.1; the corresponding conserved regions span 48 bp and 113 bp, respectively. This conserved element overlaps with neither a protein-coding gene sequence nor any predicted regulatory element, but it is located approximately 121 kbp downstream of the *ELAVL2* gene and within a predicted long non-coding RNA according to the Broad Improved Canine Annotation v1 [27]. The new fine-mapped candidate locus with a protective effect against disease development was defined based on pairwise LD estimates (r^2^ ≥ 0.8) of the fine-mapping top SNP to SNPs on CFA11. The previously identified 8.9 Mbp region was narrowed down to a 4.18 Mbp region (38,538,785 – 42,717,190) (Fig. 2a) and was confirmed as driven by one independent signal by the conditional analysis performed on the fine-mapped region (Fig. S5). The new fine-mapped candidate locus harbors 23 protein-coding genes according to the improved canine genome annotation [27]. As reported by the canine RefSeq annotation [28], it also includes 5 type I Interferon genes, potentially attractive candidates (Fig. 2b).

### Structural variation analysis identifies an association signature within the type I interferon gene cluster

In the samples that were resequenced at high coverage, CNVnator [29] predicted a total of 114 CNVs located in the fine-mapped 4.18 Mbp associated region (Table S3). After applying the stringent filtering criteria described in the Methods (section “Copy number variation (CNV) analysis”), only three CNVs were retained as being considered reliable (Table 1).

**Table 1:** CNVs predicted by CNVnator that passed the stringent filtering criteria. The CNV Del2 is shared between the HC control and HC case1, whereas two CNVs (Del1 and Del3) are private in HC case1 and the HC control respectively. No CNVs passed the filtering in HC case2. *P*-value: *p*-value of the mean normalized read depth value difference from genomic average; q0: fraction of reads mapped with mapping quality equal to zero

CNV ID	Sample	Type of CNV	CFA11 coordinates	***P***-value	q0
Del1	HC case1	deletion	38,625,401 - 38,626,900	4.8 × 10^−4^	0
Del2	HC control	deletion	40,202,601 - 40,204,100	7.5 × 10^−5^	0
Del2	HC case1	deletion	40,202,301 - 40,204,100	9.0 × 10^−6^	0
Del3	HC control	deletion	40,858,901 - 40,862,600	4.3 × 10^−11^	0

In order to confirm the predicted CNVs, LC cases and controls were screened for differences in read depth (RD) in a total of 74,216 windows on CFA11, whereas 634 windows were subsequently removed due to the low coverage. A Bonferroni corrected statistically significant (*p*-value = 1.8 × 10^− 7^) difference in coverage between the LC case and control groups was overlapping with a CNV detected by CNVnator (Del3) (CFA11: 40,858,901 - 40,862,600 bp, estimated size: ~ 3.7 kbp) (Fig. 2c). The coverage of the two groups statistically significantly deviated in a window with coordinates CFA11: 40,861,094 - 40,862,094. Nevertheless, 88 windows had a nominal *p*-value ranging from 6.8 × 10^− 7^ and 0.001, with 11 of them being located in the fine-mapped associated region. Moreover, out of these 11 windows, 3 were consecutive and overlapping with the CNV predicted by CNVnator (Del3). Figure 2d shows the fold coverage differences between the LC case and control groups based on M-values (see Methods, section “Copy number variation (CNV) analysis”) in the CNV-overlapping windows. It is worth noting that, according to M-values, the CNV might start upstream of the predicted Del3. However, in this upstream region CNVnator predicted the presence of potential CNVs that were discarded either because of overlap with a gap or repetitive sequences, which leads to zero mapping quality reads (Table S3).

The predicted Del3 is present in two copies only in the HC control, which is homozygous for the protective haplotype previously defined through GWA analysis and shows zero coverage in the region of the predicted deletion. All but one LC control samples are heterozygous for the fine-mapped protective locus and have RD consistently reduced by approximately 50% compared with LC cases in the windows overlapping Del3 (Fig. 2). We therefore concluded that the CNV is associated with the identified protective haplotype, thus representing a potential functional variant that confers protection against hypothyroidism in this dog breed. Moreover, it is striking that Del3 consistently segregates with the fine-mapping top SNP genotypes in all the whole-genome resequenced samples, despite being located ~ 1.5 Mbp upstream (Table S4). The putative CNV maps to the type I Interferon (IFN) gene family cluster and, according to the canine RefSeq annotation [28], overlaps with the potential promoter, 5′ UTR and first protein coding codons of the *IFNA7* gene.

### The deletion overlapping two predicted interferon alpha genes emerges as a plausible functional candidate

**Fig. 3:**
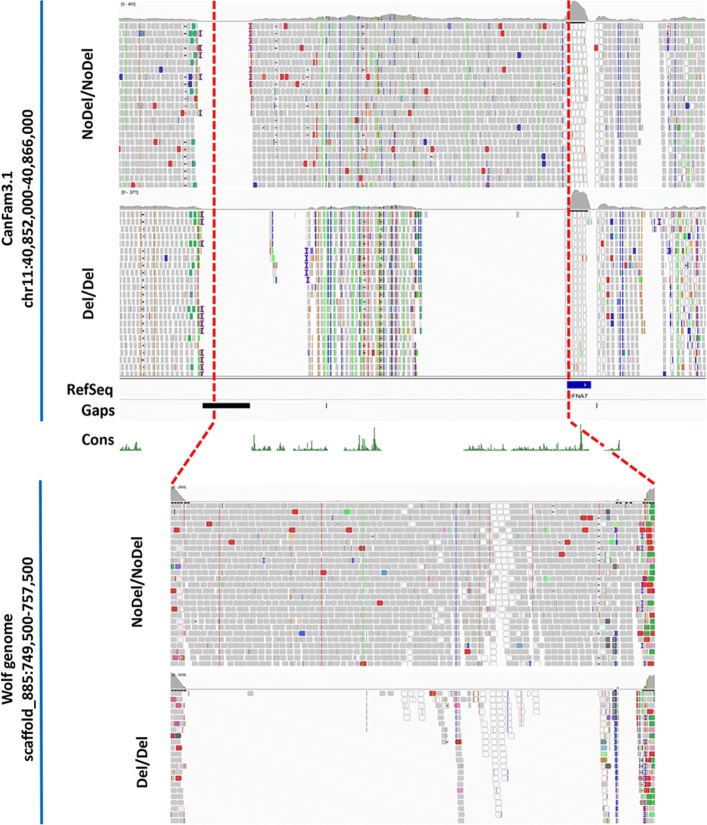
The upper panel shows the alignments of a single HC individual case (NoDel/NoDel) and the HC control (Del/Del) to CanFam3.1, including the corresponding RefSeq annotation (RefSeq), genomic gaps (Gaps) and a measure of evolutionary conservation in dog, human, mouse and rat, based on a phylogenetic hidden Markov model (phastCons) (Cons) [31]. The red dashed lines indicate the predicted deletion, with its potential start in a gap (CFA11: 40,853,967 - 40,855,084) and its end in the *IFNA7* gene (CFA11: 40,862,587 - 40,863,150), based on CanFam3.1 annotation. The prediction of the deletion location is based on the alignments of the same HC individuals (NoDel/NoDel and Del/Del) to the wolf genome, as shown in the bottom panel. The deletion is located in correspondence of a region in the scaffold_885, and more specifically between two regions with increased coverage, which are likely to be *IFNA* genes sequences

**Fig. 4:**
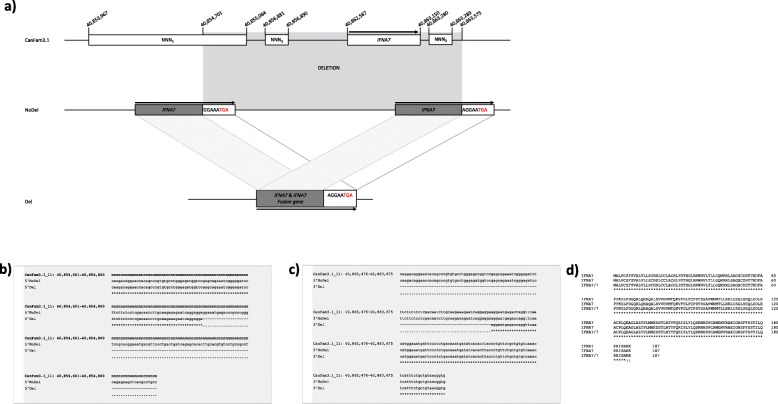
**a** The genomic organization and sequence surrounding the deletion. A comparison of canine genome assembly (CanFam3.1), the allele without the deletion (NoDel) and with deletion (Del). The deletion (chr11: 40,854,701 - 40,863,575) is indicated as a grey area and starts in a genomic gap (NNN_1_, location chr11: 40,853,967 - 40,855,084) and ends around a region of *IFNA7* (RefSeq annotation, location chr11: 40,862,587 - 40,863,150) and another genomic gap (NNN_3_, location chr11: 40,863,280 - 40,863,289). The correct location of *IFNA7* was determined based on alignment of human *IFNA7* gene to the improved canine sequence produced using Oxford Nanopore MinION sequencing. The grey dashed lines between NoDel and Del alleles indicate high sequence similarities in the deletion 5’and 3′ breakpoints. The deletion creates a fusion gene between an unannotated *IFNA* gene (*IFNA?)* and *IFNA7*, with an intact coding sequence encoding a protein identical to IFNA7 and being regulated by regulatory elements upstream *IFNA?*. The arrows indicate the transcriptional direction of the genes. **b**, **c** Multiple sequence alignments of 5′ and 3′ deletion breakpoints for the CanFam3.1 genome assembly and alleles with deletion (Del) and without deletion (NoDel). The 5′ deletion breakpoint is located in the gap on CanFam3.1 assembly, indicated with ‘n’s. **d** Protein alignments of IFNA?, IFNA7 and IFNA?/IFNA7 fusion proteins

Considering the indications that the predicted Del3 may start further upstream of the estimated start, we subsequently defined the deletion coordinates by aligning the HC case and control sequences to the wolf genome [30]. In the wolf genome, we identified the scaffold 885 (scaffold_885) as the one including the Del3 region and predicted the deletion to be significantly longer (~ 8.2 kbp), with potential start in the gap (CFA11: 40,853,967 - 40,855,084) and end within the *IFNA7* gene (CFA11: 40,862,587 - 40,863,150) annotated in the canine genome (Fig. 3). By combining long-range PCR and Oxford Nanopore MinION sequencing of the PCR products from an individual heterozygous for the deletion, we determined the exact size of the deletion to 8875 bp (CFA11: 40,854,701 - 40,863,575), including three genomic gaps in the CanFam3.1 genome assembly (Fig. 4a). Scanning for coding regions from alleles with and without the deletion indicated the presence of single exon genes with high sequence similarities to Interferon alpha (*IFNA*) genes. The deletion breakpoints were located inside two neighboring predicted *IFNA* genes (5′ breakpoint in an unannotated *IFNA* and 3′ breakpoint in the RefSeq annotated *IFNA7*) with high sequence similarity, creating a fusion gene encoding a protein identical to the one encoded by the *IFNA7* located around the deletion end (Fig. 4b-d). However, the fusion *IFNA* is missing its potential regulatory upstream elements and instead gains the putative regulatory elements from the unannotated *IFNA* gene located at the beginning of the deletion. The *IFNA* located around the deletion start is biologically missing from the individuals with the deletion. Therefore, the deletion emerges as a plausible functional candidate eliminating one *IFNA* gene and potential regulatory elements of another *IFNA* gene.

Screening for the presence of the deletion in the Giant Schnauzer study cohort identified a statistically significant enrichment of the deletion allele in the control group compared to the case group (*p*-value = 0.0001, OR = 0.17, CI_OR_ = 0.06–0.46). However, the deletion does not appear to perfectly co-segregate with either the fine-mapping top SNP (CFA11: 42,382,440) or the GWAS top SNP (CFA11: 40,777,312), as confirmed by its less significant *p*-value (Table S5). The discrepancies in allelic loads and the corresponding power of associations shown in Table S5 might be due to technical dissimilarities during genotyping experiments caused by the extreme complexity of the target region’s genomic landscape, the variable number of imputed genotypes characterizing the examined variants and their final corresponding accuracy, as well as to differences in the models used for the individual statistical analyses, respectively. We also screened wolves from four different countries ((Sweden (*n* = 2), Estonia (*n* = 2), Croatia (*n* = 1) and USA (*n* = 3)) and representatives for 17 additional dog breeds (*n* = 76) (Table S6) for the deletion. As expected, none of the wolves and the majority of the additional dog breeds did not show the deletion. However, out of seven Leonberger individuals, we identified four individuals heterozygous and two homozygous for the deletion. Unfortunately, we did not have any information on thyroid status in these dogs and can thereby only postulate the potential protective effect of the variant in the breed where hypothyroidism does occur [32].

## Discussion

In this study we identified a locus on CFA11 associated with protection against development of canine hypothyroidism in a Swedish cohort of Giant Schnauzer dogs. After performing a GWA analysis using a mixed model approach, we detected an associated locus (top SNP *p*-value = 9.9 × 10^− 6^) spanning 8.9 Mbp and conferring protection against the disease. The small sample size of our GWA analysis is likely to have hampered the statistical power of our study, as reflected by the magnitude of the association. However, the stringent phenotypic inclusion criteria used in this study may counterbalance the small sample size, the resulting level of association and its reliability, similarly to previous studies mapping canine complex traits [4, 33]. Moreover, the small sample size of our study population may have contributed to the unexpectedly long associated locus reported here. According to Lindblad-Toh and colleagues [34], the average haplotype length within a dog breed was predicted to be approximately 1 Mbp. Unexpectedly long haplotypes, such as the hypothyroidism protective locus identified in this study, could be explained by the putative causative mutation being positively selected for together with an additional desirable variant, a mechanism called hitchhiking. An alternative potential explanation could be that the putative protective haplotype appeared recently in the Giant Schnauzer breed, and has not yet undergone sufficient recombination events causing LD decay. Even though such high levels of LD allow the initial genetic mapping of a disease trait using a limited number of markers and individuals [34, 35], this genomic feature might eventually constrain the identification of the causative variant(s).

We previously described a susceptibility locus on CFA12 associated with canine hypothyroidism in three different high-risk dog breeds, the Gordon Setter, Hovawart and the Rhodesian Ridgeback [1]. The top SNP (CFA11: 40,777,312), tagging the protective locus identified in our current GWA study, is segregating in the three high-risk breeds described above and overall does not show differences in allele frequency between case and control dogs. Furthermore, based on the fixation of the hypothyroidism (non-protective) allele in the wolf population studied by Axelsson and colleagues, we postulate that this allele (allele C) represents the ancestral allele (http://genome.ucsc.edu, public track hub: Broad Improved Canine Annotation v1, track: Axelsson SNPs) [27, 36]. It is therefore plausible that this variant (CFA11: 40,777,312) appeared at a time point after domestication and before current breed creation events, considering the lack of evidences indicating gene flow between the above-mentioned breeds. Conversely, the multi-breed risk tagging variant (CFA12: 5,039,806) previously reported in three breeds [1], does not only segregate, but also shows a higher MAF in Giant Schnauzer controls compared to cases (MAF cases = 0.15; MAF controls = 0.29). This could either reflect recombination events between this risk tagging variant and the actual causative allele in the Giant Schnauzer dogs, or the absence of the causative risk allele in this breed.

Since the protective locus on CFA11 identified in this study could hide the effect of this causative risk allele, we calculated the tagging variant MAF after removing all the dogs with the protective allele on CFA11 (14 controls and 3 cases). The MAF of the tagging risk variant did not significantly change in either cases or in controls, suggesting that this locus does not contribute to hypothyroidism susceptibility in the Giant Schnauzer, thus implicating alternative disease risk loci yet to be discovered. Hence, we strengthen the hypothesis of a complex etiology underlying this disease in the existing domestic dog population, whereas there may be a few loci contributing to disease susceptibility within each breed.

By genotyping a large number of selected SNPs detected by WGS we narrowed down the associated protective candidate locus to a ~ 4 Mbp region. The prioritized variants were breed-specific and potentially causative, thus being instrumental in our fine-mapping approach, as neither the Giant Schnauzer nor any other Schnauzer breed was included in the design of the 170 K Illumina SNPChip. Such high-throughput regenotyping of selected relevant variants has previously been proven as an efficient approach for fine-mapping of genome-wide loci of association [3, 37], even though the detection of the actual causative variant(s) has been challenging. The studies that succeeded in identifying causative mutation(s) could employ an additional dog breed sharing the same candidate locus in order to pinpoint the shared minimal haplotype [38]. However, this is not an option in our study at the present time.

The CNV analysis detected a putative structural event (Del3, CFA11: 40,858,901 - 40,862,600) segregating with the genotype of the fine-mapping top SNP (CFA11: 42,382,440) in all the resequenced samples. After conclusively refining its genomic location (CFA11: 40,854,701 - 40,863,575), this deletion was found to be co-segregating with the fine-mapping top SNP in 95% (102 of 107) of the Giant Schnauzers used for the association study (94% in cases (67 of 71) and 97% in controls (35 of 36)). Moreover, it was detected in the Leonberger breed, which is prone to develop hypothyroidism. Therefore, although we could not a priori exclude the possibility of the fine-mapping top SNP being the putative causative mutation, the hypothesis of the identified CNV as the causative variant would certainly be plausible, given that conditioning for its genotype eliminates the association on CFA11 due to the strong LD. Moreover, the hypothesis of a CNV as the causative variant would be very attractive, especially considering the structural variants’ ability to reshape the gene/genomic landscape, as well as to potentially modulate gene expression. Furthermore, it is well established that CNVs contribute to phenotypic variation and disease susceptibility both in domestic animals [39–42] and humans [43–45].

According to RefSeq annotation [28], the most plausible causative variant bioinformatically identified in this study (Del3) overlaps with the region spanning both the potential promoter, 5′ UTR and first protein coding codons of the *IFNA7* gene, coding for the IFNA7 cytokine. However, we could identify the breakpoints of the deletion as located in two different *IFNA* genes, namely an unannotated *IFNA* (*IFNA?*) and the RefSeq annotated *IFNA7*. The deletion creates an *IFNA?*/*IFNA7* intact in-frame fusion gene with the coding sequence identical to the RefSeq annotated *IFNA7,* but the potential regulatory elements acquired from the *IFNA?*.

IFN-α proteins belong to type I IFNs, which are cytokines playing a major role in protecting the body from viral infections and in regulating the activity of effector immune cells [46]. In humans, this gene family has been associated with autoimmune hypothyroidism and increased serum type I IFN activity has been detected in patients with autoimmune thyroid disease [47]. Moreover, type I IFNs, particularly IFN-α, have also recently emerged as key molecules in the etiology of systemic lupus erythematosus (SLE), which is regarded as the prototype systemic autoimmune disease [48–52], thus confirming type I IFNs’ involvement in general autoimmunity. A number of studies have shown a high incidence of hypothyroidism in IFN-α-treated patients with either breast cancer [53] or hepatitis C virus infection [54]. Similarly, pre-existing thyroid autoimmunity has been shown to exacerbate in response to IFN-α treatment [55]. On top of that, the molecular mechanisms by which IFN-α triggers thyroid autoimmunity have been suggested to involve a series of complex and integrated cellular events eventually leading to the disruption of thyrocytes [56–58]. Based on the above-mentioned evidences and the hypothesis that type I IFNs can boost autoimmunity by altering the function of the immune system effector cells [59], the deletion characterized in this study emerges as a plausible candidate for protection against canine hypothyroidism. Such deletion could either remove or recruit regulatory elements that might alter the time- and tissue-specific expression of *IFNA*, thus possibly causing the protected phenotype.

Type I *IFNA* genes are organized in a cluster of paralogous genes and characterized by high levels of sequence identity, which notably complicates their assembly and annotation. The intronless nature of these genes has further contributed to overlapping and non-conclusive assignment of canine *IFNA* orthologous genes according to RefSeq annotation, and potential reference genome assembly issues. Moreover, the recent improved canine genome annotation (CanFam3.1) failed to annotate the *IFNA* gene cluster and left many gaps open in the associated locus [27] (http://genome.ucsc.edu, public track hub: Broad Improved Canine Annotation v1). In our Illumina short-read resequencing data, many reads with a reduced or zero mapping quality aligned to the genomic region surrounding *IFNA7*, thus suggesting ambiguous alignments and confirming the repetitive nature of this gene cluster. Moreover, we could not find paired-reads alignments with insert size greater than expected (indicative of a deletion), suggesting additional rearrangements or reference assembly complications.

Due to high genomic complexity in the region, high sequence similarity between single exon genes (*IFNA*) and presence of numerous gaps in the reference sequence, we were unable to determine the exact annotation of the *IFNA* genes located at the deletion breakpoints. As a consequence, we were not able to perform in silico comparative analyses of the regulatory elements presumably abolished by the deletion. Exploring valuable publicly available data (e.g. ENCODE, Epigenomics RoadMap) for the corresponding associated region in the human genome would have likely provided us with potential mechanistic interpretations of our study results [60, 61]. Undoubtedly, resequencing approaches using short-read sequencing technologies of regions with high complexity such as the *IFNA* gene cluster are unable to resolve their genomic complexities. Thus, the use of alternative long-read sequencing approaches is advisable, especially in conjunction with the improvement of the assembly and the annotation of the corresponding reference genome. Resequencing bacterial artificial chromosome (BAC) clones, WGS using long-read technology, as well as genome optical mapping would thus be desirable follow-up studies to resolve these issues [62].

## Conclusions

Using an integrative approach of methodologies ranging from genome-wide association analysis to long-read sequencing, we detected a structural variant overlapping the *IFNA* gene cluster and associated with a decreased risk of developing hypothyroidism in a high-risk dog breed. However, we were neither able to assign a specific function nor a definitive annotation to our candidate variant due to the extremely high complexity of the associated genomic region.

We also detected an association of an evolutionarily conserved SNP with protection against development of hypothyroidism and its high linkage to the candidate structural variant, which makes it extremely arduous to bridge the gap between genetic association and the revelation of the actual causative functional variation(s).

Nevertheless, the knowledge gained in this study might contribute to the development of breeding strategies, via the adoption of a marker-assisted selection eventually increasing the frequency of the candidate protective allele(s) in the population. Furthermore, our results corroborate the important role of type I IFN genes as candidates in autoimmunity and present the dog as a suitable animal model for the corresponding human diseases.

## Methods

### Study samples and phenotyping

Blood and serum samples from privately owned Giant Schnauzer dogs were collected using EDTA (1,8 mg EDTA/ml) and serum vacutainer tubes in collaboration with licensed veterinarians throughout Sweden after obtaining owners’ written approval. Samples were collected according to local ethical standards (Swedish Animal Ethical Committee No. C139/9 and C2/12 and Swedish Animal Welfare Agency No. 31–4714/09 and 31–998/12). Genomic DNA (gDNA) was extracted and serum obtained as described previously [1]. gDNA concentration was measured by NanoDrop ND-1000 Spectrophotometer and Qubit 2.0 Fluorometer (ThermoFisher Waltham, MA, USA). The proportion of fragmented gDNA was assessed by 1% agarose gel electrophoresis using 100 ng of gDNA.

For all samples used in this study we determined serological concentrations of thyroid stimulating hormone (TSH) and free thyroxine (fT4), as well as the autoantibody against thyroglobulin (TgAA). TSH and fT4 concentrations were detected using Siemens IMMULITE Immunoassay System [24, 25], whereas TgAA assay was carried out by an enzyme-linked immunosorbent assay (ELISA) [21, 63].

Dogs were classified as cases or controls based on predetermined diagnostic criteria (Table 2) in accordance with previous studies conducted by our group [24, 26]. Moreover, we excluded cases as well as controls with additional immune-related conditions based on a follow-up examination of clinical records and/or questionnaires answered by dog owners.

**Table 2:** Diagnostic criteria used to classify case and control dogs. TgAA, autoantibody against thyroglobulin; TSH, thyroid stimulating hormone; fT4, free thyroxine

Phenotype	Diagnostic criteria
Case	TgAA POS and/or TSH ≥ 40 mU/l
Control	TgAA NEG, TSH ≤ 25 mU/l, fT4 ≥ 5 pmol/l, age ≥ 7 years

### Genotyping and quality control

A sample set comprising of 115 individuals (73 cases and 42 controls) was genotyped using the Illumina 170 K CanineHD BeadChip (Illumina, San Diego, CA, USA). Chromosomal positions of SNPs are based on the dog CanFam3.1 genome assembly [27]. R v3.0.2 [64] and GenABEL v1.8–0 [65] were used in all QC steps described below. Outliers identified in an MDS plot, as well as duplicated samples and samples with sex discrepancies, were removed in a first individual-based QC step. The MDS plot can be used to show individual genetic distances deriving from a genomic kinship matrix weighted by allele frequencies and computed by using all pruned autosomal markers. In a second, marker-based QC, the total set of SNPs was in fact pruned according to MAF threshold (< 0.05), SNP and individual call rates (< 95%), *p*-values (< 1 × 10^− 3^) and false discovery rate for Hardy-Weinberg equilibrium (HWE) (< 0.2 only in controls). Furthermore, the dataset was checked for correlation between disease status and sex distribution [66, 67].

### Genome-wide association analysis

We performed a GWA analysis on the pruned dataset resulting from the individual- and marker-based QC procedures using R v3.0.2 [64] and GenABEL v1.8–0 [65]. We calculated a new genomic kinship matrix weighted by allele frequency by employing all pruned autosomal markers. Such genomic kinship matrix was also used to perform MDS in order to project and visualize the genetic distances among the pruned set of individuals in two dimensions. To identify differences in allele frequencies between cases and controls, we used a standard linear mixed model (mmscore function) that was fitted using the polygenic_hglm function from the hglm package ver 2.0–8 [68]. Considering that cases and controls shared the same geographical origin, as well as appeared as a homogenous population and uniformly interspersed in the MDS two-dimensional space after the removal of the outliers, we used a linear mixed model including genomic kinship as random effect, without the inclusion of any population-defining vector as fixed effect. We consequently used the mixed model to account for the cryptic relatedness between the individuals and their inherent population structure [69].

The statistical significance of the obtained results from the GWA analysis was evaluated as described previously [1]. Briefly, the association was defined as statistically significant if it exceeded 95% empirical SNP distributions confidence intervals (CI_95_) or an empirical genome-wide significance threshold calculated after 1000 permutations [70].

A QQ plot was constructed using R v3.0.2 [64] and a Manhattan plot generated using the R package qqman [71]. The candidate locus was defined based on pairwise LD estimates (r^2^ ≥ 0.8) of the most significantly associated SNP (top SNP) to the rest of the SNPs on the chromosome. The independence of the association signal was tested by a conditional analysis in which the genotype of the top SNP was included as a covariate in the statistical model. Conditional analysis determines the independence of additional association signals from the leading top SNP and the variants in LD with it compared to the remaining variants in the candidate locus.

### Whole-genome resequencing

We performed WGS for individuals representing the key haplotypes as detected by the GWA analysis. One control individual homozygous for the protective allele (control) and two cases homozygous for the non-protective allele (case1 and case2) were sequenced at high coverage (> 40X). Additionally, 10 controls heterozygous for the protective allele and 10 cases homozygous for the non-protective allele were sequenced at low coverage (< 10X). Sequencing libraries were sequenced as paired-end reads (2 × 101 bp) with HiSeq2500 (Illumina, San Diego, CA, USA) by using the services of the National Genomics Infrastructure at Science for Life Laboratory, Stockholm, Sweden.

The resulting reads were mapped to the dog genome assembly CanFam 3.1 using the Burrows-Wheeler aligner (BWA) v0.6.2-r126 [72]. The software Picard v1.64 was utilized for marking PCR duplicates and for evaluating alignment quality (http://broadinstitute.github.io/picard). Base quality recalibration and local realignment were performed using the Genome Analysis Toolkit (GATK) v1.5–11-g5c5d8e7 [73]. Variant calling was performed within the GATK framework using UnifiedGenotyper, and the identified polymorphisms were hard-filtered according to standard parameters [74].

For the following analyses, only SNPs with differing genotypes between the HC control and the HC cases were included. The SNPs were annotated using SnpEFF [75] and their effect also predicted using the Variant Effect Predictor (VEP) webtool (http://www.ensembl.org/vep). In order to target potential causative mutations and to restrict the number of SNPs suitable for subsequent genotyping and fine-mapping, we sought to categorize the SNPs according to different criteria. We prioritized SNPs based on the determined annotation and the predicted effect, as well as their overlap with either conserved elements according to 29 mammals conservation scores [76] or regions of promoters, protein coding and antisense sequences according to the public track hub Broad Improved Canine Annotation v1 (http://genome.ucsc.edu) [27]. Moreover, we used Integrative Genome Viewer (IGV) [77] to confirm the calling reliability of the resulting set of prioritized SNPs.

### SNP genotyping, imputation and fine-mapping

Sequenom MassARRAY technology (http://www.sequenom.com/iplex) was employed to regenotype the selected subset of SNPs in the majority of samples previously included in the GWA analysis (*n* = 96). The regenotyped SNPs were subsequently phased and imputed in the few missing samples (*n*_cases_ = 8, *n*_controls_ = 3) using Beagle v3.0 [78, 79], as well as employing a reference dataset comprising of the Illumina SNPChip (see Methods, section “Genotyping and quality control”) and Sequenom MassARRAY regenotyped SNPs, in which variants were pruned based on MAF < 0.001. We subsequently performed a two-step filtering removing SNPs with imputation likelihood lower than empirically defined thresholds. Firstly, SNPs with an allelic squared correlation (R^2^) value lower than 0.75 were discarded. Secondly, genotypes with imputation probability values lower than 0.8 were labeled as missing. The filtered imputed data were merged with the Illumina SNPChip and regenotyping data produced in previous steps, in order to obtain a comprehensive dataset including all the study samples and all genotyped SNPs. We then performed fine-mapping of the determined genome-wide associated region using GenABEL [65] with the quality control steps, statistical model, conditional analysis and LD estimation procedures described above.

### Copy number variation (CNV) analysis

In order to detect additional potentially causative variants, we employed the software CNVnator (ver 0.3) [29] to scan the whole chromosome of interest for potential CNVs. By using default options and a 100 bp bin size, we sought to detect read depth (RD) differences between the HC cases and the HC control. CNVnator predicts genomic structural variations (deletions/duplications) based on RD, while correcting for GC-content. We filtered CNV calls in the fine-mapped region of association by the mean RD value difference from genomic average (*p*-value < 0.001) and the length of the CNV (> 1000 bp). Moreover, we only retained CNV calls without any reads with zero mapping quality (q0 filter). Finally, we discarded all CNV calls overlapping with gaps in the reference genome. The CNVs predicted in the samples sequenced at high coverage were examined in the 10 LC cases and 10 LC controls.

It has previously been shown that, despite yielding overall accurate results, CNVnator might fail to detect CNVs in low coverage samples [29]. For this reason, we employed an alternative approach to examine the LC cases and controls. Firstly, we included only reads with mapping quality ≥15 and extracted the RD for every position on the chromosome of interest using the GATK function DepthOfCoverage [73]. Secondly, we calculated normalized RD in 1 kbp non-overlapping windows by using in-house perl scripts. All samples were normalized by applying a correction factor based on the sample showing the highest average coverage (LC case1, see Table S1) to each window. Windows with the coverage lower than 2X in every sample were excluded from the following analysis. We then performed a t-test to detect statistically significant depth differences between the case and the control groups for each defined window. The Bonferroni corrected threshold (*p*-value < 6.8 × 10^− 7^, corrected for the number of tests) was used to define statistically significant coverage differences between the two groups of samples. In addition, we computed M-values as following:

$$ M=\mathit{\log}2\left(\frac{window\ depth}{case\ group\ window\ mean\ depth}\ \right) $$

M-values represent the fold-coverage differences between the LC case and control groups. M-values were calculated in each sample for every window overlapping with the CNV regions shared between all resequenced samples. RD and M-values were plotted and visualized by using R v3.0.2, and the corresponding deviating genomic regions examined in IGV [77] and UCSC genome browser [80].

### CNV definition and genotyping

The detected CNV (deletion) with functional potential was further defined by alignment of HC cases and HC control to the wolf genome [30], using the mem algorithm of BWA v0.7.12 [72]. The wolf scaffold corresponding to the canine region of interest was identified by mapping relevant canine anchor sequences to the wolf genome using blast [81] and the alignments visualized using IGV [77].

For amplification across the region of interest, long-range PCR primers (Table S7) were designed using Primer3 v.0.4.0 [82, 83], PCR performed using PrimeSTAR GXL DNA Polymerase (TaKaRa Bio, Osaka, Japan) and long-fragment DNA prepared using MagAttract HMW DNA Kit (Qiagen AB, Sollentuna, Sweden), following manufacturer’s instructions. Long-range PCR products (estimated size ~ 14 and 6 kbp) were sequenced with an Oxford Nanopore MinION sequencer using a R9.4.1 pore flow cell, with a barcoded library generated using the LSK108 kit and the native barcoding kit according to the manufacturer’s instructions (Oxford Nanopore Technologies, UK). The deletion coordinates were determined from alignments of MinION sequencing data to the CanFam 3.1 genome assembly using multiple sequence alignment (MAFFT v.7) [84]. The potential coding regions were predicted from both alleles using GENSCAN [85].

In the individuals with available DNA (*n* = 101), genotyping of the deletion was performed using a three-primer approach (Fig. S6, Table S7), with primers designed as above and PCR performed using AmpliTaq Gold DNA Polymerase (Thermo Fisher Scientific, Waltham, MA, USA) following the manufacturer’s instructions and using the elongation time optimal for ~ 1 kbp. The PCR products (NoDel = 906 bp, Del = 1038 bp) were size-separated using 2.5% agarose gel. In the remaining individuals lacking DNA specimen (*n* = 6), the deletion genotype was imputed with the same method as described earlier (see Methods, section “SNP genotyping, imputation and fine-mapping”). The reference dataset for this imputation comprised of the PCR-typed deletion genotypes, along with the Illumina SNPChip and Sequenom MassARRAY regenotyped SNPs covering the extended region of association. Fisher’s exact test was used to determine whether the deletion allele frequency was statistically significantly (*p*-value < 0.05) different between the Giant Schnauzer case and control dogs.

## Supplementary information

**Additional file 1: Figure S1.** (a) MDS plot showing the sample set before quality control (QC). The red circle highlights the outlier samples (*n* = 6). The black arrow indicates the outlier sample (*n* = 1) with the standard coat color. (b) MDS plot showing the sample set after quality control (QC).

**Additional file 2: Figure S2.** (a) QQ plot showing the observed versus expected SNPs *p*-value distribution. After the mixed model approach, the inflation factor λ is equal to 0.93. The QQ plot also shows the empirical genome-wide significance threshold (indicated by a red line and its corresponding –log10 value equal to 5.14) and empirical 95% confidence intervals (CI_95_) (indicated by solid grey lines). (b) Manhattan plot showing a peak of association on CFA11 (*p*-value_raw_ = 9.9 × 10^− 6^).

**Additional file 3: Figure S3.** Manhattan plot after conditioning the GWA analysis for the top SNP genotype.

**Additional file 4: Figure S4.** (a) QQ plot showing the observed versus expected SNPs *p*-value distribution of the final complete dataset including both GWA and fine-mapping SNPs. After the mixed model approach, the inflation factor λ is equal to 0.97. The QQ plot also shows the empirical genome-wide significance threshold, *p*-value = 5.4 × 10^− 6^ (indicated by a red line and its corresponding –log10 value equal to 5.27), and empirical 95% confidence intervals (CI_95_) (indicated by solid grey lines). (b) Manhattan plot confirming the detection of a peak of association on CFA11 (*p*-value_raw_ = 5.7 × 10^− 6^) during the fine-mapping experiment.

**Additional file 5: Figure S5.** Manhattan plot after conditioning the GWA analysis for the fine-mapping top SNP genotype.

**Additional file 6: Figure S6.** The 3-primer design for deletion genotyping. Primers NF11 and NR12 give a PCR product (906 bp) from only the allele without the deletion. Primers NF3 and NR12 give PCR products from both alleles without the deletion (~ 11,000 bp) and with the deletion (1038 bp). However, using the PCR elongation optimal for amplifying up to 1 kbp produced only the two shorter fragments (906 and 1038 bp), enabling the genotype determination during the subsequent separation on the agarose gel.

**Additional file 7:** **Table S1.** Table showing the genotypes of the GWA analysis top SNP (CFA11: 40,777,312), average coverage and proportions of reads mapping to Canfam3.1 for the sequenced HC and LC case (highlighted in dark and light red) and HC and LC control (highlighted in dark and light blue) samples. HC: high coverage; LC: low coverage.

**Additional file 8: Table S2.** Number of SNPs that were successfully pooled for high-throughput re-genotyping in all the samples (Pooled Variants), number of SNPs that were subsequently genotyped with success (Successfully genotyped variants), and criteria for variant selection (Functional Category): Conserved elements (SNPs overlapping conserved elements with SiPhy LOD-score higher than 7 based on 29 mammals conservation scores), VEP (SNPs predicted to have an effect on the amino acid sequence according to the Variant Effect Predictor webtool analysis), Antisense/Protein coding transcripts (SNPs overlapping predicted antisense and protein coding transcripts), Promoter (SNPs overlapping predicted promoters of genes located in the associated genomic region), AF difference (SNPs with high allele frequency differences between LC cases and LC controls), SNPChip (control SNPs included in the Illumina SNPChip for the genotype concordance check between the two experiments), Fill the gaps (SNPs located in the regions of low coverage in the extended region of association).

**Additional file 9 Table S3.** Table showing all CNVs detected in the fine-mapped region of association by CNVnator. For every CNV the following information is displayed: sample showing the CNV (Sample); type of CNV (Event); genomic coordinates (Chr:start-stop); length of the CNV (Event Length); CNV normalized read depth (Normalized RD); *p*-value of the mean normalized read depth value difference from genomic average (*p*-val1); *p*-value from probability of read depth values within the region to be in the tails of Gaussian distribution describing frequencies of read depth values in bins (*p*-val2); same as *p*-val1, but for the middle of the CNV (*p*-val3); same as *p*-val2, but for the middle of the CNV (p-val4); fraction of reads mapped with mapping quality equal to zero (q0).

**Additional file 10: Table S4.** Table showing the genotypes of the GWA study top SNP (CFA11: 40,777,312), the predicted CNV (Del3) inferred genotypes (CFA11: 40,858,901 - 40,862,600) and genotypes of the fine-mapping top SNP (CFA11: 42,382,440) of the sequenced HC and LC case (highlighted in dark and light red) and HC and LC control (highlighted in dark and light blue) samples. HC: high coverage; LC: low coverage.

**Additional file 11: Table S5.** Association *P*-value, number of imputed genotypes, number of detected protective and risk alleles both in cases (*n* = 71) and controls (*n* = 36) for the GWAS top SNP, fine mapping top SNP and the deletion associated with protection to hypothyroidism (DELETION).

**Additional file 12: Table S6.** Overview of all individuals genotyped for the deletion.

**Additional file 13: Table S7.** Primer sequences, used annealing temperatures and times and predicted PCR product lengths used in the study.

## Data Availability

All the whole-genome sequence data generated in the study were deposited in the European Nucleotide Archive (ENA) (https://www.ebi.ac.uk/ena) under the accession number: PRJEB35554. All the genotype and phenotype files are available from the Dryad database (doi:10.5061/dryad.0rxwdbrvq).

## Abbreviations

BAC: Bacterial artificial chromosome
BWA: Burrows-Wheeler aligner
CI_95_: 95% empirical SNP distribution confidence intervals
CLT: Canine lymphocytic thyroiditis
CNV: Copy number variation
DLA: Dog leukocyte antigen
ELISA: Enzyme-linked immunosorbent assay
fT4: Free thyroxine
GATK: Genome Analysis Toolkit
gDNA: Genomic DNA
GWA: Genome-wide association
HC: High coverage
HT: Hashimoto’s thyroiditis
HWE: Hardy-Weinberg equilibrium
IFN: Interferon
IFNA: Interferon alpha
IGV: Integrative Genome Viewer
LC: Low coverage
LD: Linkage disequilibrium
MAF: Minor allele frequency
MDS: Multidimensional scaling
OR: Odds ratio
QC: Quality control
QQ: Quantile-quantile
R^2^: Allelic squared correlation
RD: Read depth
SD: Standard deviation
SLE: Systemic lupus erythematosus
T3: Triiodothyronine
T4: Thyroxine
TgAA: Autoantibody against thyroglobulin
TSH: Thyroid stimulating hormone
VEP: Variant Effect Predictor
WGS: Whole-genome resequencing
