# Supplementary figures and images for: Whole-genome genotyping and resequencing reveal the association of a deletion in the complex interferon alpha gene cluster with hypothyroidism in dogs
Source: BMC Genomics. 2020 Apr 16;21:307. doi: 10.1186/s12864-020-6700-3 (PMC7160888; doi:10.1186/s12864-020-6700-3)

a)

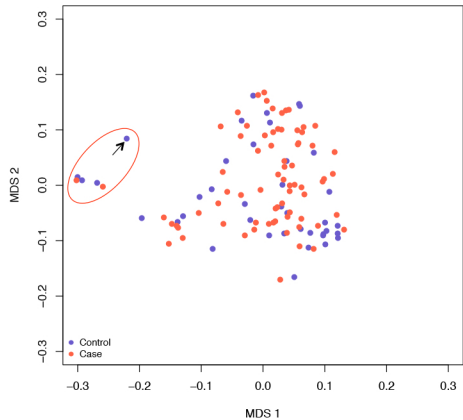

b)

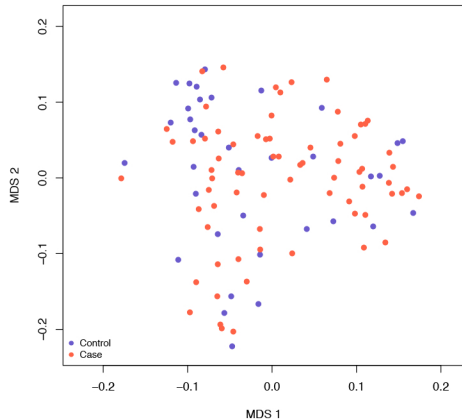

Supplement: Supplementary file 1 — Additional file 1: Figure S1. (a) MDS plot showing the sample set before quality control (QC). The red circle highlights the outlier samples (n = 6). The black arrow indicates the outlier sample (n = 1) with the standard coat color. (b) MDS plot showing the sample set after quality control (QC). [file 12864_2020_6700_MOESM1_ESM.pdf]

a)

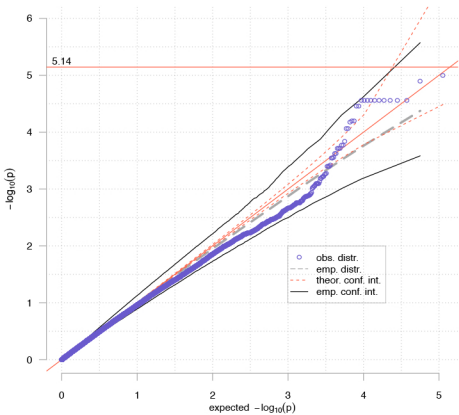

b)

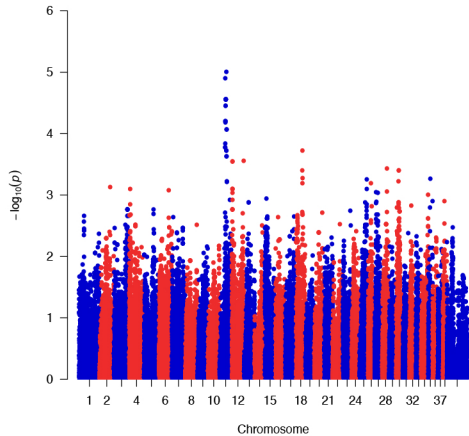

Supplement: Supplementary file 2 — Additional file 2: Figure S2. (a) QQ plot showing the observed versus expected SNPs p-value distribution. After the mixed model approach, the inflation factor λ is equal to 0.93. The QQ plot also shows the empirical genome-wide significance threshold (indicated by a red line and its corresponding –log10 value equal to 5.14) and empirical 95% confidence intervals (CI95) (indicated by solid grey lines). (b) Manhattan plot showing a peak of association on CFA11 (p-valueraw = 9.9 × 10− 6). [file 12864_2020_6700_MOESM2_ESM.pdf]

$-\log_{10}(\text{P-value})$

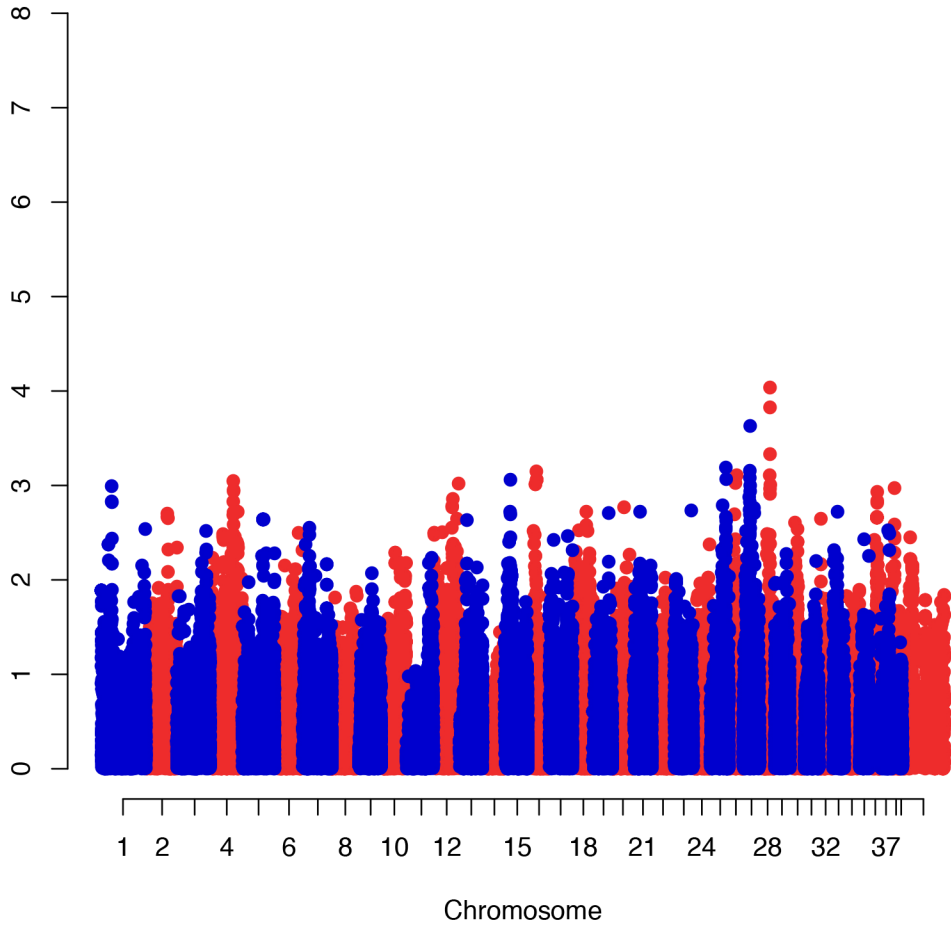

Supplement: Supplementary file 3 — Additional file 3: Figure S3. Manhattan plot after conditioning the GWA analysis for the top SNP genotype. [file 12864_2020_6700_MOESM3_ESM.pdf]

a)

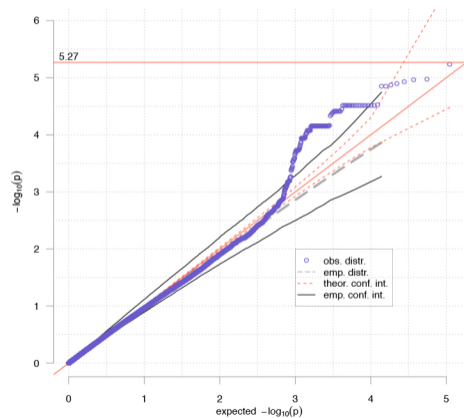

b)

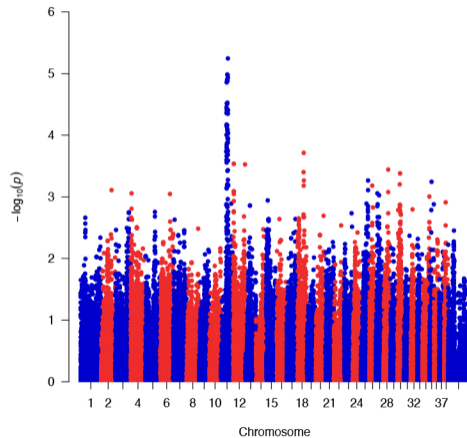

Supplement: Supplementary file 4 — Additional file 4: Figure S4. (a) QQ plot showing the observed versus expected SNPs p-value distribution of the final complete dataset including both GWA and fine-mapping SNPs. After the mixed model approach, the inflation factor λ is equal to 0.97. The QQ plot also shows the empirical genome-wide significance threshold, p-value = 5.4 × 10− 6 (indicated by a red line and its corresponding –log10 value equal to 5.27), and empirical 95% confidence intervals (CI95) (indicated by solid grey lines). (b) Manhattan plot confirming the detection of a peak of association on CFA11 (p-valueraw = 5.7 × 10− 6) during the fine-mapping experiment. [file 12864_2020_6700_MOESM4_ESM.pdf]

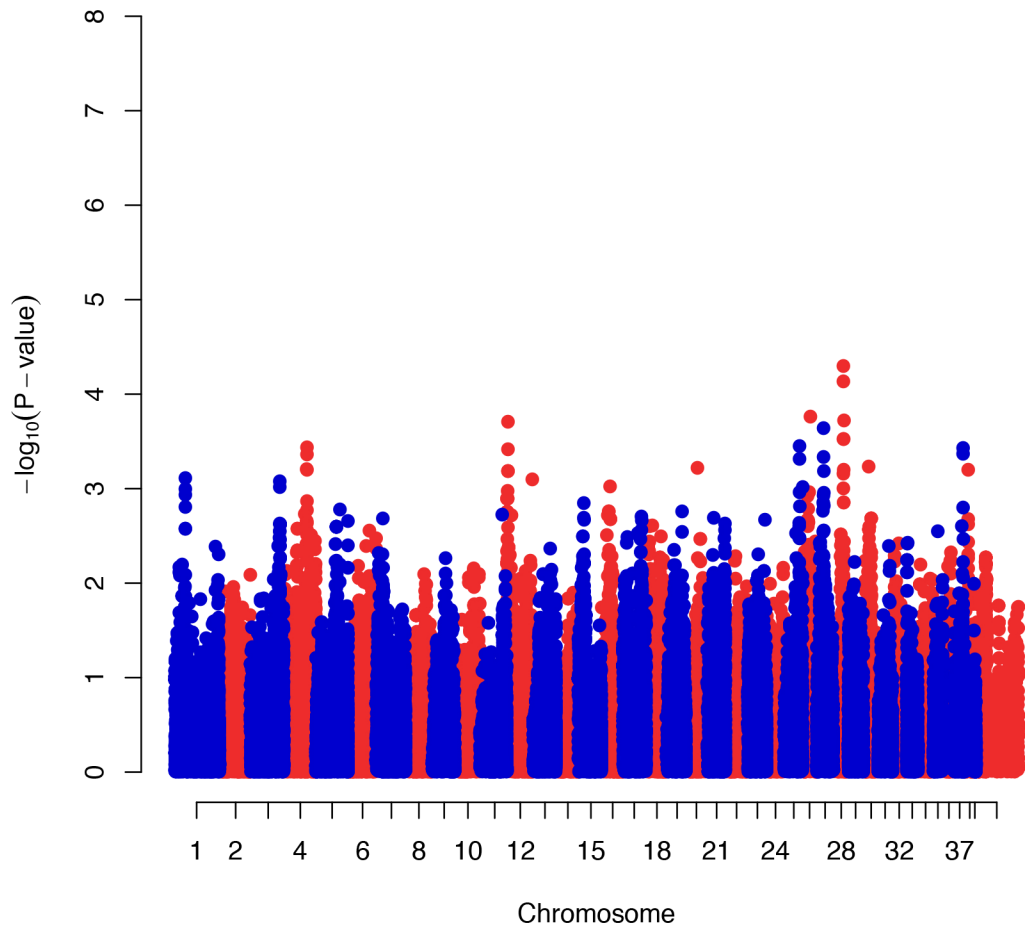

Supplement: Supplementary file 5 — Additional file 5: Figure S5. Manhattan plot after conditioning the GWA analysis for the fine-mapping top SNP genotype. [file 12864_2020_6700_MOESM5_ESM.pdf]

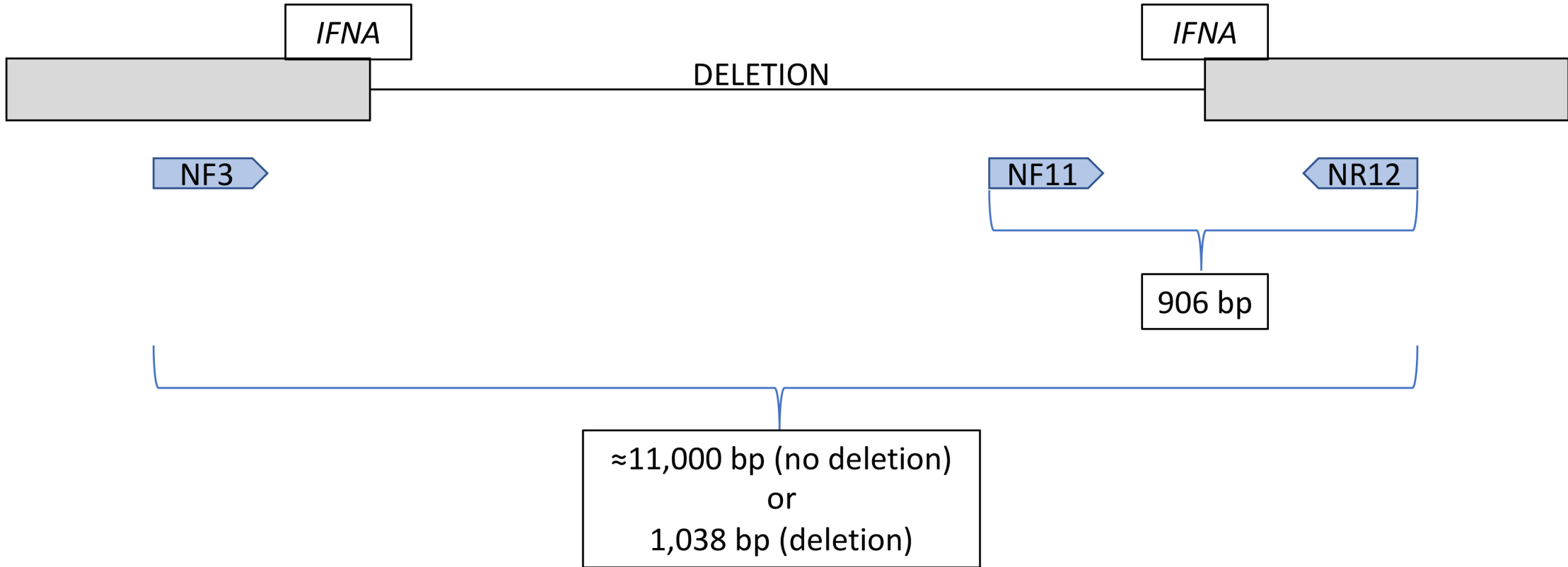

Supplement: Supplementary file 6 — Additional file 6: Figure S6. The 3-primer design for deletion genotyping. Primers NF11 and NR12 give a PCR product (906 bp) from only the allele without the deletion. Primers NF3 and NR12 give PCR products from both alleles without the deletion (~ 11,000 bp) and with the deletion (1038 bp). However, using the PCR elongation optimal for amplifying up to 1 kbp produced only the two shorter fragments (906 and 1038 bp), enabling the genotype determination during the subsequent separation on the agarose gel. [file 12864_2020_6700_MOESM6_ESM.pdf]
