# Supplementary material for: Whole-genome genotyping and resequencing reveal the association of a deletion in the complex interferon alpha gene cluster with hypothyroidism in dogs
Source: BMC Genomics. 2020 Apr 16;21:307. doi: 10.1186/s12864-020-6700-3 (PMC7160888; doi:10.1186/s12864-020-6700-3)
Supplement: Supplementary file 8 — Additional file 8: Table S2. Number of SNPs that were successfully pooled for high-throughput re-genotyping in all the samples (Pooled Variants), number of SNPs that were subsequently genotyped with success (Successfully genotyped variants), and criteria for variant selection (Functional Category): Conserved elements (SNPs overlapping conserved elements with SiPhy LOD-score higher than 7 based on 29 mammals conservation scores), VEP (SNPs predicted to have an effect on the amino acid sequence according to the Variant Effect Predictor webtool analysis), Antisense/Protein coding transcripts (SNPs overlapping predicted antisense and protein coding transcripts), Promoter (SNPs overlapping predicted promoters of genes located in the associated genomic region), AF difference (SNPs with high allele frequency differences between LC cases and LC controls), SNPChip (control SNPs included in the Illumina SNPChip for the genotype concordance check between the two experiments), Fill the gaps (SNPs located in the regions of low coverage in the extended region of association). [file 12864_2020_6700_MOESM8_ESM.docx]

**Table S2**

| **Functional Category** | **Pooled Variants** | **Successfully genotyped variants** |
| --- | --- | --- |
| Conserved elements | 481 | 464 |
| VEP | 77 | 72 |
| Antisense/Protein coding transcripts | 75 | 72 |
| Promoter | 90 | 84 |
| AF difference | 6 | 6 |
| SNPChip | 5 | 4 |
| Fill the gaps | 6 | 5 |
| **TOTAL** | **740** | **707** |

Number of SNPs that were successfully pooled for high-throughput re-genotyping in all the samples (Pooled Variants), number of SNPs that were subsequently genotyped with success (Successfully genotyped variants), and criteria for variant selection (Functional Category): **Conserved elements** (SNPs overlapping conserved elements with SiPhy LOD-score higher than 7 based on 29 mammals conservation scores), **VEP** (SNPs predicted to have an effect on the amino acid sequence according to the Variant Effect Predictor webtool analysis), **Antisense/Protein coding transcripts** (SNPs overlapping predicted antisense and protein coding transcripts), **Promoter** (SNPs overlapping predicted promoters of genes located in the associated genomic region), **AF difference** (SNPs with high allele frequency differences between LC cases and LC controls), **SNPChip** (control SNPs included in the Illumina SNPChip for the genotype concordance check between the two experiments), **Fill the gaps** (SNPs located in the regions of low coverage in the extended region of association).
