# Supplementary material for: Whole-genome genotyping and resequencing reveal the association of a deletion in the complex interferon alpha gene cluster with hypothyroidism in dogs
Source: BMC Genomics. 2020 Apr 16;21:307. doi: 10.1186/s12864-020-6700-3 (PMC7160888; doi:10.1186/s12864-020-6700-3)
Supplement: Supplementary file 11 — Additional file 11: Table S5. Association P-value, number of imputed genotypes, number of detected protective and risk alleles both in cases (n = 71) and controls (n = 36) for the GWAS top SNP, fine mapping top SNP and the deletion associated with protection to hypothyroidism (DELETION). [file 12864_2020_6700_MOESM11_ESM.docx]

**Table S5.**

|  |  | **GWAS TOP SNP** | **FINE-MAPPING TOP SNP** | **DELETION** |
| --- | --- | --- | --- | --- |
|  | **ASSOCIATION P-VALUE** | 9.9x10^-6^ | 5.7x10^-6^ | 1.1x10^-4^ |
|  | **SAMPLE SIZE** | 107 | 107 | 107 |
|  | **IMPUTED GENOTYPES** | 0 | 11 (8 cases, 3 controls) | 6 (3 cases, 3 controls) |
|  |  |  |  |  |
| **CASES** | **PROTECTIVE ALLELE** | 7 | 3 | 7 |
|  | **RISK ALLELE** | 135 | 139 | 135 |
| **CONTROLS** | **PROTECTIVE ALLELE** | 19 | 16 | 17 |
|  | **RISK ALLELE** | 53 | 56 | 55 |

**Association P-value, number of imputed genotypes, number of detected protective and risk alleles both in cases (n=71) and controls (n=36) for the GWAS top SNP, fine mapping top SNP and the deletion associated with protection to hypothyroidism (DELETION).**
